# Enhancing Quercetin Bioavailability Attenuates Aging Phenotypes via the Gut Microbiota–Intestinal Barrier Axis in Aged Mice

**DOI:** 10.3390/nu18101537

**Published:** 2026-05-12

**Authors:** Yuji Naito, Katsura Mizushima, Ryo Inoue, Tomohisa Takagi

**Affiliations:** 1 Department of Human Immunology and Nutrition Science, Graduate School of Medical Science, Kyoto Prefectural University of Medicine, Kamigyo-ku, Kyoto 602-8566, Japan; mizusima@koto.kpu-m.ac.jp; 2 Molecular Gastroenterology and Hepatology, Graduate School of Medical Science, Kyoto Prefectural University of Medicine, Kamigyo-ku, Kyoto 602-8566, Japan; takatomo@koto.kpu-m.ac.jp; 3 Laboratory of Animal Science, Department of Applied Biological Sciences, Faculty of Agriculture, Setsunan University, Osaka 573-0101, Japan; ryo.inoue@setsunan.ac.jp

**Keywords:** aging, bioavailability, quercetin, gut microbiota, senescence

## Abstract

Background/Objectives: Aging is characterized by progressive functional decline associated with alterations in gut microbiota, epithelial barrier dysfunction, and cellular senescence. Although quercetin has been proposed as a potential anti-aging compound, its clinical application is limited by poor bioavailability. In this study, we investigated whether enhancing quercetin bioavailability using EubioQuercetin (EQN) modulates aging-related phenotypes through the gut microbiota–intestinal barrier axis. Methods: Male C57BL/6J mice were treated with EQN or conventional quercetin (CQN) for 12 weeks. External aging phenotypes were assessed using a composite aging score based on hair glossiness, hair loss, and the presence of white hair. Gut microbiota composition was analyzed via 16S rRNA sequencing with centered log-ratio transformation, and intestinal gene expression was assessed by quantitative reverse transcription-polymerase chain reaction. Results: EQN significantly reduced the aging score compared with the control group (median 4.5 vs. 8, *p* < 0.01), while CQN also showed a moderate reduction. Microbiota analysis identified taxa positively associated with aging (*Lactobacillus*, *Romboutsia*, *Desulfovibrio*, and *Lachnoclostridium*) and negatively associated taxa (*Akkermansia* and *Christensenellaceae*). EQN suppressed aging-associated taxa and partially increased taxa linked to a healthier microbiota profile. At the intestinal level, EQN downregulated senescence-associated genes (*p21*, *PCNA*, and *Lgr5*) and upregulated the tight junction gene *claudin-1*. In contrast, systemic inflammatory markers and short-chain fatty acids were not significantly associated with the aging score. Conclusions: These findings indicate that enhancing quercetin bioavailability attenuates externally assessed aging phenotypes in aged mice and is associated with coordinated changes in gut microbiota and intestinal gene expression. Modulation of the gut microbiota–intestinal barrier axis may represent a potential mechanism underlying these effects.

## 1. Introduction

Aging is a complex biological process characterized by the progressive accumulation of cellular and molecular damage, leading to a decline in physiological functions, increased disease susceptibility, and ultimately mortality. The World Health Organization defines aging as a gradual loss of intrinsic capacity over time [1]. In recent years, the emerging field of geroscience has focused on identifying the fundamental mechanisms that drive aging and developing interventions that can simultaneously delay or prevent multiple age-related diseases.

Among the key hallmarks of aging, chronic low-grade inflammation, often referred to as “inflammaging,” and gut microbiota alterations are central contributors [2]. The gut–microbiota axis possibly plays a pivotal role in regulating systemic aging processes by interacting with host metabolism, immune function, and epithelial homeostasis. Specific microbial taxa, including *Akkermansia muciniphila* and members of the *Christensenellaceae* family, are associated with healthy aging and metabolic resilience [3].

Cellular senescence is another fundamental driver of aging and age-related diseases. Senolytic therapies that selectively eliminate senescent cells have yielded promising results in preclinical models [4]. Particularly, the combination of dasatinib and quercetin has been demonstrated to reduce tissue inflammation, improve metabolic function, and modulate immune responses in aged animals [5,6,7]. Recent studies have also shown that senolytic interventions alleviate intestinal senescence and inflammation while reshaping the gut microbiota composition [8]. These findings highlight the close interplay among cellular senescence, intestinal homeostasis, and the microbiome in the regulation of aging.

Quercetin, a widely distributed dietary polyphenol, has attracted considerable attention owing to its anti-inflammatory, antioxidant, and senolytic properties [9]. However, its clinical application remains limited by low water solubility and bioavailability, resulting in insufficient systemic exposure when administered orally. To overcome these limitations, EubioQuercetin (EQN), a highly water-soluble quercetin derivative, has been developed [10,11]. EQN is derived from isoquercetin (quercetin-3-O-glucoside), a glycosylated form with improved water solubility and intestinal absorption compared with quercetin aglycone. In addition, EQN incorporates proprietary formulation technology to enhance bioavailability [11]. Pharmacokinetic and microbiological studies have demonstrated that EQN exhibits superior absorption and bioavailability compared to conventional quercetin (CQN), suggesting that it exerts enhanced biological effects [11].

Bioavailable flavonoids exert protective effects against age-related dysfunctions [12], including cognitive decline [10], supporting the potential of improved polyphenol formulations for aging interventions. Based on these considerations, we hypothesized that EQN exerts stronger anti-aging effects than CQN via enhanced modulation of the gut microbiota–intestinal axis. To verify this, the present study aimed to evaluate the effects of EQN on external aging phenotypes in aged mice and elucidate the underlying mechanisms via integrated analyses of the gut microbiota composition, intestinal gene expression, and systemic proteomics.

## 2. Materials and Methods

### 2.1. Animals and Experimental Design

Thirty male C57BL/6J mice (age: 48 weeks; Oriental Yeast Co., Ltd., Tokyo, Japan) were purchased for this study. C57BL/6J mice were selected because this strain is widely used in aging-related and metabolic studies and exhibits well-characterized age-associated phenotypic changes, including alterations in hair condition, intestinal physiology, and gut microbiota composition. At the start of the experiment, the mean body weight of the animals was 35.8 + 0.7 g. After a two-week acclimation period, the mice were randomly assigned to three groups (*n* = 10/group) using a computerized randomization program (IBUKI, Japan Bio Research Center, Gifu, Japan) to ensure comparable mean body weight and variance among groups. Sample size was determined based on feasibility and consistency with previous exploratory animal studies of quercetin-related interventions [10,11]. All animals were housed in a controlled environment under the following conditions: 20.0–26.0 °C temperature, 40.0–70.0% humidity, a 12 h light/dark cycle (lights on from 6:00 to 18:00), and ventilation at 12 air changes per hour. The animals were housed five per cage in polycarbonate cages (dimensions: 30 × 20 × 13 cm) containing wood chip-based bedding, which was changed regularly under standard hygienic conditions. Autoclaved tap water was provided ad libitum using water bottles throughout the experimental period. The animals were monitored daily for general health status, and body weight was measured weekly. The control (CON) group received a standard AIN93G diet. The EQN group received AIN93G supplemented with EQN (ALPS Pharmaceutical Industry Co., Ltd., Gifu, Japan), whereas the CQN group received AIN93G supplemented with quercetin (ALPS Pharmaceutical Industry Co., Ltd.). All diets were provided ad libitum for 12 weeks. Food intake was measured weekly throughout the study. KM alone knows the group allocation at the different stages of the experiment.

The experiments were conducted in accordance with the NIH guidelines for the use of animals in research [13]. The animal experiment protocol was reviewed and approved by the Ethics Committee for Animal Experiments established at the Japan Bio-Research Center (No. 430276, 27 February 2024).

### 2.2. Dose Calculation

The dose of EQN was set at 150 mg/kg/d based on previous studies [10,11]. Assuming that each mouse consumed approximately 5 g of diet per day, with 70% ingested and 30% lost, the dietary concentration of EQN was adjusted to 0.12% (1.2 g/kg diet). This resulted in an estimated daily intake of 4.2 mg per mouse, corresponding to 150 mg/kg/d for a 28 g mouse. To ensure molar equivalence, the concentration of CQN (molecular weight: 338) was adjusted to 0.033% based on the molecular weight of rutin (molecular weight: 664). The human equivalent dose, calculated using a conversion factor of 12.3, was approximately 800 mg/d.

### 2.3. Aging Score Assessment

Aging phenotypes were evaluated based on external physical characteristics using a standardized scoring system. Digital images of the face (frontal view) and dorsum were obtained using a digital camera at baseline and on days 27, 55, and 83 after treatment initiation. For facial imaging, the mice were gently restrained in a cylindrical holder to ensure consistent positioning. Three parameters were evaluated: Hair glossiness, hair loss, and the presence of white hair. Each parameter was scored independently for the face and dorsum using the following criteria:

#### 2.3.1. Hair Glossiness

- Score 0: Clearly glossy (normal condition)
- Score 1: Slightly reduced gloss
- Score 2: Clearly reduced gloss
- Score 3: Markedly reduced gloss
- Score 4: Severely deteriorated and dirty appearance

##### Hair Loss

- Score 0: No hair loss
- Score 1: Hairless area < 25% and thinning < 50%
- Score 2: Hairless area < 25% and thinning ≥ 50%
- Score 3: Hairless area = 25–50%
- Score 4: Hairless area > 50%

##### The Presence of White Hair

- Score 0: No white hair
- Score 1: White hair detectable upon close inspection
- Score 2: Clearly visible white hair
- Score 3: Diffuse white hair

To minimize observer bias, all evaluations were performed in a blinded manner with respect to group allocation. Scoring was conducted according to standardized criteria to ensure consistency across animals. Although this scoring system was developed specifically for the present study, it provides a quantitative and reproducible measure of externally observable aging-related changes. The total aging score was calculated as the sum of the individual scores.

### 2.4. Functional Assessments

#### 2.4.1. Rotarod Test

Motor coordination was assessed using a rotarod apparatus (UGO BASILE, Gemonio, Italy), with the rotation speed accelerating from 5 to 40 rpm over 5 min [14]. The latency to fall was recorded, with a maximum cut-off time of 300 s. Each mouse was tested thrice at intervals of at least 30 min.

#### 2.4.2. Wire Hang Test

The mice were placed on an inverted wire grid, and the latency to fall was recorded, with a maximum cut-off time of 600 s [15]. Each mouse underwent two trials at intervals of at least 60 min.

#### 2.4.3. Y-Maze Test

Spontaneous alternation behavior was assessed using the Y-maze apparatus [16]. The mice were allowed to explore freely for 8 min. Subsequently, the total number of arm entries and the spontaneous alternation percentage were determined.

### 2.5. Sample Collection

At the end of the 12-week treatment period (day 84), mice were deeply anesthetized with isoflurane, and blood samples were collected from the inferior vena cava using ethylenediaminetetraacetic acid-coated syringes. Euthanasia was performed by exsanguination via total blood collection under deep anesthesia. Death was confirmed by cessation of heartbeat and respiratory movement prior to tissue collection [17]. Plasma was separated via centrifugation (4 °C, 2200× *g*, 15 min) and stored at −80 °C. After exsanguination, the small intestine, cecal contents, and skeletal muscles were collected. Intestinal samples were cleaned with saline, and cecal contents were stored for microbiota and metabolite analyses.

### 2.6. Gut Microbiota Analysis

Gut microbial DNA extraction, library preparation, and MiSeq sequencing were performed as previously described [18]. Briefly, microbial DNA was extracted and purified from 25 mg of cecal contents using the QuickGene DNA Tissue Kit SII (KURABO, Osaka, Japan). This microbial DNA was used to amplify the V3–V4 region of *16S rRNA* using the primer sets 341F (5′-CCTACGGGNGGCWGCAG-3′) and 805R (5′-GACTACHVGGGTATCTAATCC-3′) [13]. Polymerase chain reaction (PCR) was conducted according to the following program: Initial denaturation at 95 °C for 3 min, followed by 25 cycles of 95 °C for 30 s, 55 °C for 30 s, and 72 °C for 30 s, and a final extension step at 72 °C for 5 min. Amplicons were purified using NucleoFast96 PCR plates (TaKaRa Bio, Kusatsu, Japan) and subjected to a second PCR with unique dual-index primer sets for MiSeq sequencing. The resulting amplicons were purified using the SequalPrep Normalization Plate Kit (Life Technologies, Tokyo, Japan) and AMPure XP beads (Beckman-Coulter, Brea, CA, USA) and pooled, followed by 285 bp paired-end sequencing on the Illumina MiSeq platform (Illumina, San Diego, CA, USA) with MiSeq Reagent Kit v3. Raw sequences have been deposited in the National Center for Biotechnology Information Sequence Read Archive under the BioProject ID PRJNA1256317 (available from 1 May 2026).

Data obtained from MiSeq sequencing were analyzed using QIIME2 version 2022.2 [19]. To construct amplicon sequence variants (ASVs), paired-end reads were denoised using DADA2 with the q2-dada2 plugin [20]. Taxonomic classification of ASVs was performed using the Naive Bayes classifier with the q2-classifier sklearn plugin against the SILVA 138 99% reference dataset. Singletons and ASVs assigned to mitochondria and chloroplasts were removed from downstream analyses. A phylogenetic tree was constructed via SATé-enabled phylogenetic placement [21]. Alpha diversity indices were calculated using QIIME2 by setting the sampling depth to 5000. Outputs from QIIME2 were further analyzed using the R program (https://www.R-project.org/, accessed on 1 September 2025) with the Bioconductor packages Phyloseq [22] and MicrobiotaProcess [23]. Beta diversity was calculated based on weighted and unweighted UniFrac distances. Microbiota data were analyzed at the genus level after CLR transformation with zero replacement, followed by Z-score standardization. Statistical significance was assessed via Spearman’s rank correlation analysis, with FDR correction using the Benjamini–Hochberg method.

### 2.7. Measurement of Organic Acid Levels in Cecal Contents

To measure the levels of fecal organic acids, including acetate, propionate, iso-butyrate, butyrate, iso-valerate, valerate, succinate, lactate, and formate, 0.3 g of feces was mixed with 600 µL of distilled water and 90 µL of 14% perchloric acid and centrifuged at 13,000× *g* for 10 min at 4 °C. The supernatant was filtered through a 0.45 µm cellulose acetate membrane filter (Cosmonice Filter W; Nakalai Tesque, Kyoto, Japan) and degassed under vacuum. The resulting supernatant was subjected to organic acid measurement using a high-performance liquid chromatography apparatus with the SIL-10 autoinjector (Shimadzu, Kyoto, Japan), as previously described [24].

### 2.8. mRNA Expression Analysis

Total RNA was isolated using the acid guanidinium phenol–chloroform method with TRIzol reagent (Thermo Fisher Scientific, Waltham, MA, USA). The resultant cDNA was subjected to quantitative reverse transcription (qRT)-PCR using specific primers for six adhesion molecules (zona occludens-1, occludin, and claudin-1, -6, -7, and -8), three mucins (Muc2, Muc3, and Muc4), growth factors, and cell cycle proteins (Ki67, leucine-rich repeat-containing G protein-coupled receptor 5 [Lgr5], Bmi1, musashi RNA-binding protein 1, Hopx, proliferating cell nuclear antigen [PCNA], p57, p16, p21, and cyclin D1). All forward and reverse primers are listed in Supplementary Table S1. PCR was performed using the PowerUp SYBR Green PCR Master Mix and QuantStudio 6 Pro Real-Time PCR System (Thermo Fisher Scientific). The PCR conditions included 40 cycles of 95 °C for 15 s and primer annealing at 60 °C for 1 min, followed by a melting curve analysis in which the temperature was increased from 60 to 95 °C [25]. Gene expression levels were calculated from the qRT-PCR data relative to those of glyceraldehyde-3-phosphate dehydrogenase.

To evaluate global patterns of intestinal gene expression, principal component analysis (PCA) was performed using the mRNA expression data of genes related to barrier function, cell proliferation, and aging. Before PCA, gene expression values were standardized via Z-score transformation to ensure comparability across genes. PCA was conducted using Python (version 3.10) with the scikit-learn package (version1.8.0). The first two principal components (PC1 and PC2) were used to visualize clustering patterns among samples. To illustrate group-wise data distribution, 95% confidence ellipses were constructed for each group based on the covariance matrix of PC1 and PC2 scores. The ellipses were calculated assuming a multivariate normal distribution and scaled using the chi-square distribution with two degrees of freedom (χ^2^ = 5.991), corresponding to the 95% confidence region.

### 2.9. Measurement of Blood Biochemistry and Proteomics

Standard biochemical parameters were measured, and plasma proteomic analysis was performed using the Olink Target 48 panel including 19 interleukins, 5 chemokines, and 10 cytokines [26].

### 2.10. Statistical Analyses

All statistical analyses were conducted using Python (version 3.10; Python Software Foundation, Wilmington, DE, USA), unless otherwise specified. Data are represented as the mean ± standard deviation or median (interquartile range), as appropriate. Comparisons among the three groups (CON, EQN, and CQN) were performed using one-way analysis of variance followed by post hoc Tukey’s multiple comparison test for normally distributed variables. For non-normally distributed data, the Kruskal–Wallis test followed by Dunn’s post hoc test was applied. Spearman’s rank correlation analysis was used to evaluate the associations between aging score and microbiota abundance, gene expression levels, organic acid levels, and proteomic marker levels. To account for multiple comparisons, false discovery rate correction (FDR) was performed using the Benjamini–Hochberg method. For microbiota data, relative abundances at the genus level were transformed via centered log-ratio transformation after zero replacement (1 × 10^−6^), followed by Z-score standardization before statistical analysis. For gene expression analysis, relative mRNA expression levels were normalized to glyceraldehyde-3-phosphate dehydrogenase levels and analyzed using appropriate parametric or non-parametric tests, as described above. Statistical significance was set at *p* < 0.05, unless otherwise specified.

## 3. Results

### 3.1. Body Weight and Food Intake

During the experimental period, two mice in the EQN group died on days 4 and 9, respectively, after treatment initiation. Necropsy was performed; however, the cause of death could not be determined. No abnormal findings were observed in the remaining EQN group animals throughout the study period. Therefore, the final number of animals analyzed in the EQN group was reduced to *n* = 8, whereas the CON and CQN groups each consisted of *n* = 10 animals. No significant differences in food consumption and body weight were observed among the three groups throughout the study period (Table 1).

### 3.2. Aging Score

**Figure 1:**
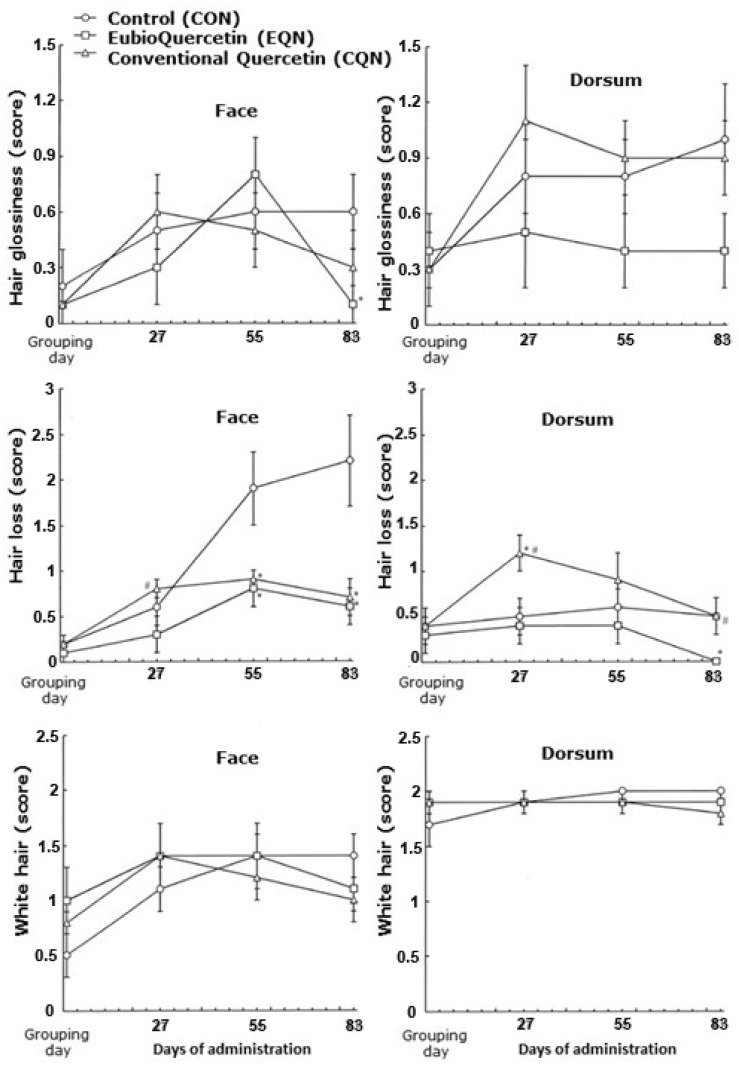
Temporal changes in external aging parameters. Changes in hair glossiness, hair loss, and the presence of white hair scores on the face and dorsum were evaluated during the experimental period. Data are represented as the mean ± standard deviation (SD). * *p* < 0.05 vs. the CON group, # *p* < 0.05 vs. the EQN group. Sample sizes were *n* = 10 for the CON and CQN groups and *n* = 8 for the EQN group.

**Figure 2:**
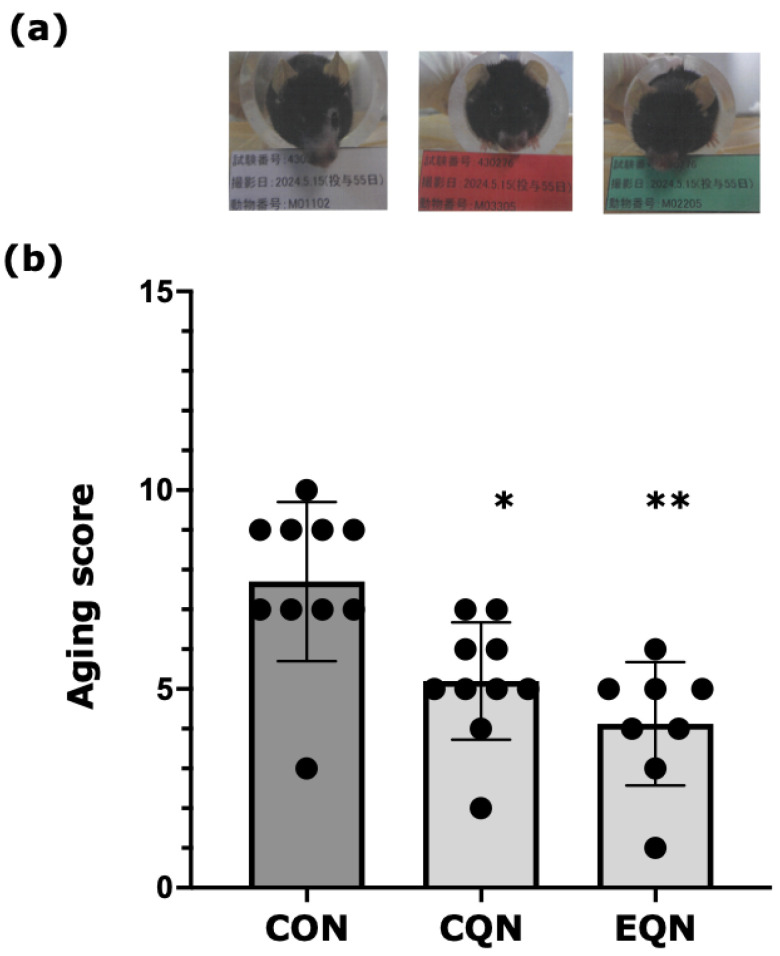
Comparison of the aging score among experimental groups. (**a**) Representative images of mice in each group. (**b**) The aging score, calculated as the sum of hair glossiness, hair loss, and the presence of white hair scores, was compared among the control (CON), conventional quercetin (CQN), and EubioQuercetin (EQN) groups. Data are represented as individual values. Statistical analysis was conducted using the non-parametric Dunn’s test. * *p* < 0.05 and ** *p* < 0.01 vs. the CON group. Sample sizes were *n* = 10 for the CON and CQN groups and *n* = 8 for the EQN group.

Temporal changes in external aging parameters, including hair glossiness, hair loss, and the presence of white hair, are shown in Figure 1. In the CON group, the hair glossiness score on the dorsum increased markedly by day 27 and remained elevated until day 83. This increase was significantly suppressed in the EQN group. The hair loss score increased more prominently on the face than on the dorsum in the CON group; however, this increase was attenuated in both the EQN and CQN groups. The white hair score showed modest changes without significant differences among groups. The composite aging score, calculated as the sum of hair glossiness, hair loss, and white hair scores, is shown in Figure 2a,b. Compared to that in the CON group (median = 8), the aging score was significantly reduced in both the CQN (median = 5; *p* < 0.05) and EQN (median = 4.5; *p* < 0.01) groups, with a greater reduction observed in the EQN group.

### 3.3. Muscle and Cognitive Functions

No significant differences in muscle strength measured by the rotarod and wire hang tests, muscle mass measured by cross-sectional area, or cognitive function measured by the Y-maze test were observed among groups (Table 1).

### 3.4. Gut Microbiota

**Figure 3:**
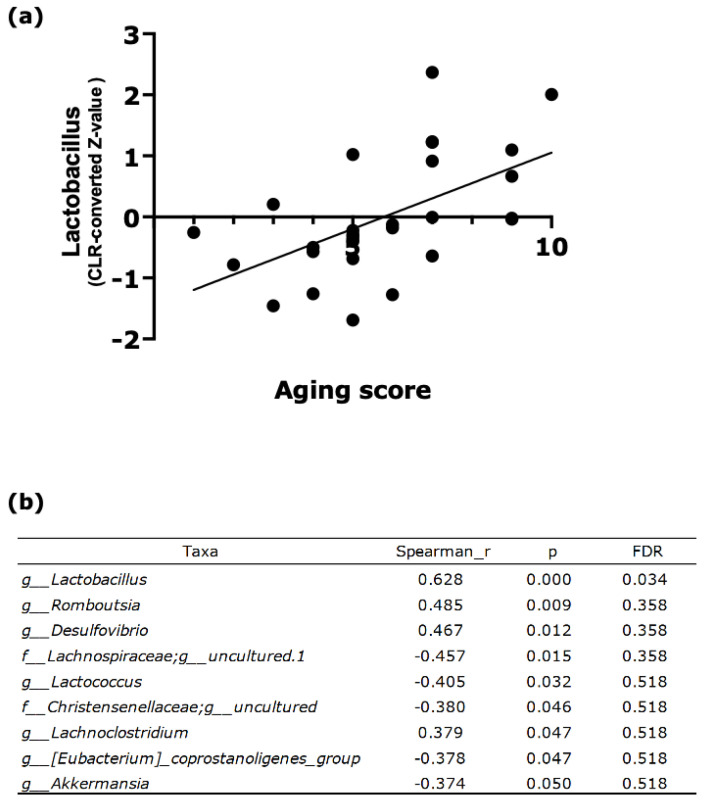
Correlation between gut microbiota abundance and the aging score. (**a**) Representative scatter plot showing the association between the aging score and *Lactobacillus* abundance (centered log-ratio [CLR]-transformed Z-score). The solid line indicates the linear regression fit. Spearman’s ρ = 0.628; *p* < 0.001; false discovery rate (FDR) = 0.034. (**b**) Spearman’s rank correlation analysis between the aging score and gut microbial taxa. Nine taxa with *p* < 0.05 were identified. Spearman’s ρ, *p*-values, and FDR-adjusted values are shown. Sample sizes were *n* = 28.

Spearman’s rank correlation analysis identified nine bacterial taxa significantly associated with the aging score (Figure 3b). Among these, *Lactobacillus*, *Romboutsia*, *Desulfovibrio*, and *Lachnoclostridium* were significantly positively correlated, whereas members of the *Lachnospiraceae* family (uncultured genus), *Lactococcus*, *Christensenellaceae* (uncultured genus), *Eubacterium coprostanoligenes group*, and *Akkermansia* were negatively correlated with the aging score. Notably, *Lactobacillus* demonstrated the strongest positive correlation with the aging score (Figure 3a, Spearman’s ρ = 0.628; *p* < 0.001; false discovery rate = 0.034), suggesting robust associations between this genus and external aging phenotypes.

**Figure 4:**
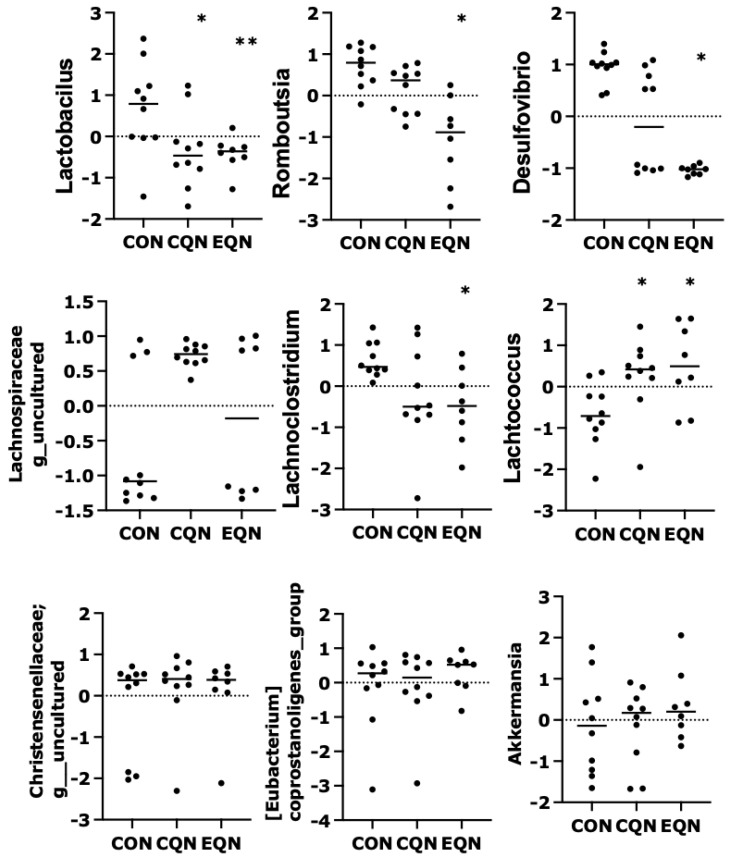
Comparison of aging-associated gut microbiota among experimental groups. Relative abundances of nine selected bacterial taxa significantly associated with the aging score were compared among the CON, CQN, and EQN groups. Data are represented as individual values (CLR-transformed Z-scores). Statistical analysis was conducted using the non-parametric Dunn’s test. * *p* < 0.05 and ** *p* < 0.01 vs. the CON group. CON, control; CQN, conventional quercetin; EQN, EubioQuercetin; CLR, centered log-ratio. Sample sizes were *n* = 10 for the CON and CQN groups and *n* = 8 for the EQN group.

To further investigate the impacts of interventions on aging-associated microbiota, the relative abundances of the nine selected taxa were compared among the three groups (CON, CQN, and EQN; Figure 4). Among the taxa positively associated with aging, *Lactobacillus* abundance was significantly reduced in both the CQN and EQN groups compared to that in the CON group. Similarly, *Desulfovibrio* abundance was significantly decreased in the EQN group. Similarly, the abundances of *Romboutsia* and *Lachnoclostridium* exhibited a trend toward reduction, with more pronounced decreases observed in the EQN group. In contrast, among the taxa negatively associated with aging, *Lactococcus* abundance was significantly increased in both the CQN and EQN groups, whereas *Akkermansia* and *Eubacterium coprostanoligenes group* abundances tended to be elevated, although these changes did not reach statistical significance. Members of the *Lachnospiraceae* and *Christensenellaceae* families showed a modest increase in abundance without significant differences among groups.

### 3.5. Organic Acid Levels in Cecal Contents

The concentrations of nine organic acids in cecal contents were measured. Notably, no organic acid showed a significant correlation with the aging score. Additionally, no significant differences in organic acid concentrations were observed among the three groups (Supplementary Table S2).

### 3.6. mRNA Expression Levels of Intestinal Genes

**Figure 5:**
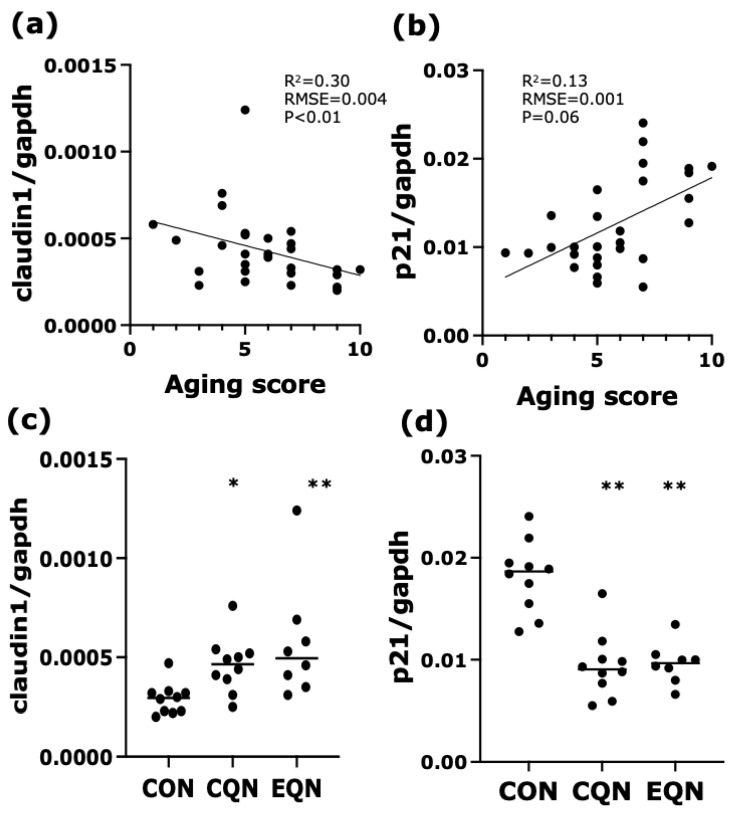
Association between intestinal gene expression and the aging score, and comparison among experimental groups. Upper panels (**a**,**b**) show correlations between the aging score and intestinal mRNA expression levels of *claudin-1* and *p21*. Linear regression lines are shown. *Claudin-1 expression* exhibited a significant inverse correlation, whereas *p21* showed a positive correlation trend with the aging score. Lower panels (**c**,**d**) show group comparisons of mRNA expression levels among the CON, CQN, and EQN groups. Data are represented as individual values and median. Statistical analysis was conducted using the non-parametric Dunn’s test. * *p* < 0.05 and ** *p* < 0.01 vs. the CON group. Sample sizes were *n* = 10 for the CON and CQN groups and *n* = 8 for the EQN group.

The mRNA expression levels of 20 genes related to intestinal barrier function, cell proliferation, and aging were quantified via RT-PCR. Spearman’s rank correlation analysis identified several intestinal genes associated with the aging score. Among these, *p21*, *PCNA*, *Lgr5*, and *Ki67* were positively correlated, whereas *claudin-1*, *claudin-6*, and *p16INK4a* were negatively correlated with the aging score (Supplementary Table S3). Notably, *claudin-1* expression showed a significant inverse correlation with the aging score (R2 = 0.30; *p* < 0.01), indicating strong associations between intestinal barrier integrity and external aging phenotypes. In contrast, *p21* expression exhibited a positive correlation trend with the aging score (R2 = 0.13; *p* = 0.06; Figure 5a,b). Comparisons among groups revealed that *p21* levels were significantly reduced in both the CQN and EQN groups compared to those in the CON group. Conversely, *claudin-1* levels were significantly upregulated in the EQN and CQN groups (*p* < 0.01 and *p* < 0.05, respectively; Figure 5c,d).

**Figure 6:**
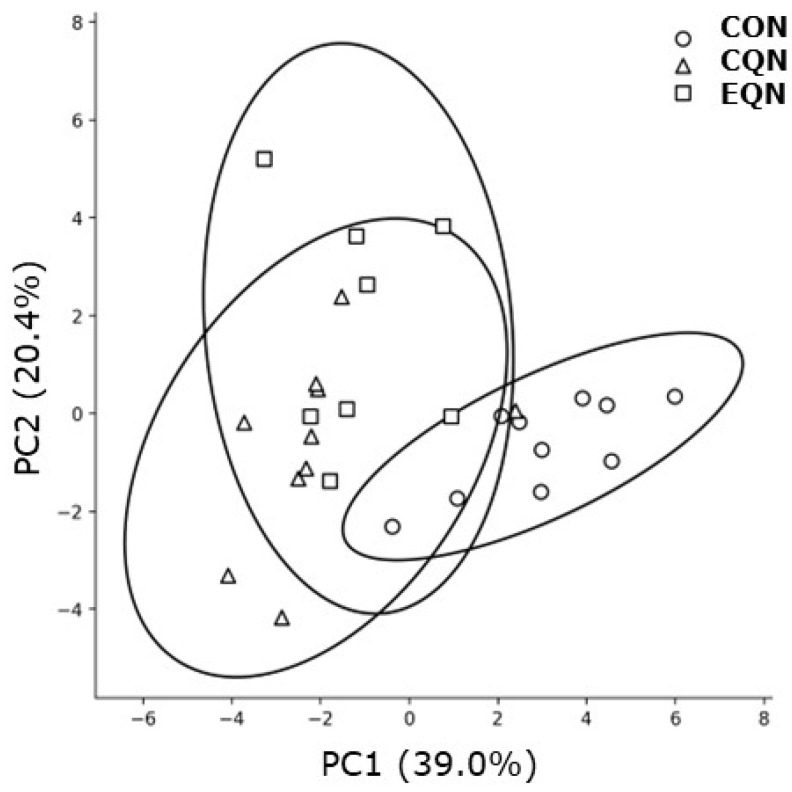
Principal component analysis (PCA) of intestinal mRNA expression with 95% confidence ellipses. Principal component analysis (PCA) was performed using intestinal mRNA expression data. Each point represents an individual mouse, and the circle, triangle, and square indicate the CON, CQN, and EQN experimental groups, respectively. Ellipses represent the 95% confidence intervals based on the covariance of each group. The first two principal components (PC1 and PC2) are shown, with the percentage of variance explained indicated on each axis. Samples from the EQN group were clearly separated from those from the CON group, indicating distinct gene expression profiles. The CQN group showed partial separation, suggesting intermediate effects. Sample sizes were *n* = 10 for the CON and CQN groups and *n* = 8 for the EQN group.

To determine whether intestinal gene expression profiles differed globally, PCA was performed using mRNA expression data. As shown in Figure 6, samples from the three groups exhibited distinct clustering patterns in the PC1–PC2 space. Notably, the EQN group was clearly separated from the CON group, indicating that EQN induced a distinct intestinal gene expression profile. The CQN group showed partial overlap with the CON group but also exhibited a tendency toward separation, indicating intermediate effects. These findings suggest that EQN both affects individual genes and globally remodels intestinal gene expression patterns. Furthermore, the separation of groups in PCA supports the notion that the coordinated regulation of barrier-related and senescence-associated genes underlies the observed anti-aging effects.

### 3.7. Blood Biochemistry and Proteomics

Notably, no abnormalities were observed in blood biochemical parameters. Proteomic analysis revealed no significant associations between most protein levels and the aging score. Only hepatocyte growth factor (HGF) levels showed a trend toward a negative correlation (r = −0.370; *p* = 0.052) and tended to increase in the CQN group.

## 4. Discussion

In this study, we demonstrated that EQN, a highly bioavailable formulation of quercetin, significantly attenuates external aging phenotypes in aged male mice. These effects were accompanied by coordinated alterations in gut microbiota composition and intestinal gene expression, suggesting that enhancement of quercetin bioavailability modulates aging-related processes through the gut microbiota–intestinal barrier axis. The occurrence of mortality in the CQN group may have affected statistical power and group balance, and therefore the findings should be interpreted with caution.

Quercetin has attracted considerable attention as a potential anti-aging compound; however, its clinical application has been limited by poor solubility and low bioavailability. Isoquercetin, a glycosylated form of quercetin, exhibits improved intestinal absorption, and EQN further enhances this bioavailability through formulation technology [11]. Our findings that EQN exerted more pronounced effects on aging score and molecular parameters than conventional quercetin (CQN) support the concept that bioavailability is a critical determinant of the biological efficacy of dietary polyphenols. This is consistent with previous pharmacokinetic and functional studies demonstrating improved absorption and biological activity of water-soluble quercetin derivatives [8].

A key finding of this study is the association between gut microbiota composition and external aging phenotype. Several bacterial taxa, including *Lactobacillus*, *Romboutsia*, *Desulfovibrio*, and *Lachnoclostridium* [27], were positively associated with aging score, whereas *Akkermansia* and members of the *Christensenellaceae* family showed negative associations [3]. Notably, EQN suppressed taxa positively associated with aging while partially increasing taxa linked to a healthier microbiota profile. Although *Lactobacillus* is generally considered beneficial, recent studies suggest that its functional role is context-dependent and may vary according to host age, microbial community structure, and strain-level differences [28]. Therefore, the observed increase in *Lactobacillus* in association with higher aging scores may reflect age-related dysbiosis rather than a universally beneficial effect.

Interestingly, despite these microbiota changes, short-chain fatty acids (SCFAs) were not significantly associated with aging score. This finding suggests that the anti-aging effects observed in this study may not be primarily mediated by global changes in microbial metabolite levels. Instead, specific microbial taxa and localized host–microbe interactions may play a more important role. This interpretation is consistent with emerging evidence that microbial composition and functional interactions, rather than bulk metabolite abundance alone, are critical determinants of host physiology.

At the intestinal level, EQN induced coordinated changes in gene expression related to epithelial senescence and barrier function. Senescence-associated genes such as *p21*, *PCNA*, and *Lgr5* were positively associated with aging score and were suppressed by EQN. In contrast, tight junction-related genes, particularly *claudin-1* [29], were negatively associated with aging score and were upregulated in the EQN group. The inverse relationship between *claudin-1* expression and aging score highlights the importance of intestinal barrier integrity in aging-related phenotypes. While *claudin-6* also showed a similar trend, its role in adult intestinal tissue is less well established and may reflect epithelial differentiation or regeneration processes rather than a primary barrier function.

The opposing regulation of senescence-associated and barrier-related genes suggests a coordinated restoration of intestinal homeostasis by EQN. Increased *p21* expression reflects epithelial senescence and impaired tissue renewal [30], whereas increased *claudin-1* expression indicates improved barrier integrity. The simultaneous suppression of *p21* and enhancement of *claudin-1* by EQN supports a model in which attenuation of epithelial senescence and reinforcement of barrier function contribute to improved external aging phenotype. These findings are in line with the concept of “gut frailty,” in which deterioration of intestinal barrier function and epithelial homeostasis contributes to systemic aging processes [31]. Collectively, our results provide experimental evidence linking intestinal barrier integrity to external aging phenotypes.

In contrast to the marked intestinal changes, systemic proteomic analysis revealed minimal associations between circulating inflammatory markers and aging score. Only hepatocyte growth factor (HGF) showed a weak negative association. Given its role in tissue repair and regeneration, this observation may reflect a compensatory response rather than a primary driver of aging. The lack of strong systemic inflammatory signals suggests that the observed anti-aging effects are predominantly mediated by local intestinal mechanisms rather than systemic inflammation. Although inflammatory cytokines such as interleukins are central to the concept of inflammaging, their contribution in this model may be limited or tissue-specific, which was not directly assessed in the present study.

The main strengths of this study include the use of aged animals, a comprehensive multi-omics approach integrating microbiota, gene expression, and proteomics, and direct comparisons with CQN. Several limitations should be acknowledged. First, this study focused on external aging phenotypes, and functional measures such as muscle performance and cognitive function did not show significant differences among groups. Therefore, the observed effects should be interpreted as improvements in externally assessed aging phenotype rather than systemic anti-aging effects. Second, causal relationships between microbiota changes and host phenotypes were not directly tested. Third, untargeted metabolomic analysis was not performed, and thus, broader metabolic alterations could not be evaluated. Fourth, the study was conducted in a relatively small number of animals, and two deaths occurred during the experimental period, which may affect statistical power and group balance. Finally, the findings are based on a single animal model and require validation in other models and in human studies. Future studies should incorporate microbiota transplantation, metabolomic profiling, and clinical trials to validate our findings and further elucidate the underlying mechanisms. In addition, this study was conducted exclusively in aged male C57BL/6J mice, and therefore the findings may not be directly generalizable to humans. Species-specific differences in aging biology, gut microbiota composition, and quercetin metabolism should be considered when interpreting these results. Further clinical studies are needed to evaluate the translational relevance of EQN in human aging.

## 5. Conclusions

In conclusion, EubioQuercetin attenuated external aging phenotypes in aged male mice and was associated with coordinated changes in gut microbiota composition and intestinal gene expression. These findings suggest that enhancing the bioavailability of dietary polyphenols modulates aging-related processes through gut-centered mechanisms, particularly via the microbiota–intestinal barrier axis. Further studies are warranted to clarify causal relationships and to evaluate the translational potential of these findings in humans.

## Figures and Tables

**Table 1:** Body weight, food consumption, muscle function, and cognitive functions among CON, CQN, and EQN groups. Sample sizes were *n* = 10 for the CON and CQN groups and *n* = 8 for the EQN group.

	Control (CON)			Conventional Quercetin (CQN)			EubioQuercetin (EQN)		
	Mean	±	SD	Mean	±	SD	Mean	±	SD
Body weight (g)									
Before grouping	35.6	±	0.8	35.9	±	0.8	35.8	±	0.8
Day 28	37.5	±	0.7	38.1	±	0.8	38.4	±	0.9
Day 56	38.7	±	0.9	39.2	±	0.9	39.2	±	1.1
Day 84	40.7	±	0.9	40.7	±	0.8	40.5	±	1.0
Food consumption (g/day/animal)									
Before grouping	2.5			2.5			2.1		
Day 29	2.8			2.6			2.4		
Day 57	2.5			2.8			2.6		
Day 84	2.4			2.4			2.3		
Muscle weight (g)									
Soleus muscle (mg)	16.2	±	0.3	16.7	±	0.5	16.8	±	0.5
Soleus muscle (mg%)	40.0	±	1.0	41.1	±	1.2	41.7	±	1.6
Plantaris muscle (mg)	37.6	±	0.3	38.1	±	0.7	35.3	±	1.0
Plantaris muscle (mg%)	92.8	±	2.3	93.7	±	2.1	87.4	±	2.5
Muscle cross-sectional area (mm^2^)									
Soleus muscle	1.01	±	0.21	0.87	±	0.18	0.94	±	0.16
Plantaris muscle	1.18	±	0.17	1.29	±	0.21	1.27	±	0.18
Rotarod test (s)									
Before grouping	106.5	±	16.2	98.5	±	16.8	105.8	±	15.0
Day 26	115.5	±	17.7	101.9	±	12.6	109.4	±	9.3
Day 54	103.8	±	6.5	97.8	±	18.6	86.6	±	11.5
Day 82	98.5	±	9.5	81	±	12	96.7	±	13.5
Wire hang test (latency to fall, s)									
Before grouping	153.2	±	30.9	220.6	±	28.2	139.4	±	25.6
Day 27	267.8	±	51.0	234.8	±	46.3	229.9	±	31.7
Day 55	204.4	±	44.2	206.1	±	42.7	176.8	±	30.4
Day 83	96.2	±	19.4	106.4	±	25.1	123.1	±	19.9
Y maze test									
Total arm entries (counts)	15.9	±	1.3	17	±	1.6	16.4	±	1.5
Spontaneous % alteration (%)	59.2	±	3.8	62.7	±	3.7	47.5	±	5.5

## Data Availability

The original contributions presented in this study are included in the article and Supplementary Materials. Further inquiries can be directed to the corresponding author (Y.N.).

## Supplementary Materials

The following supporting information can be downloaded at: https://www.mdpi.com/article/10.3390/nu18101537/s1, Table S1: Primers used for qRT-PCR assay; Table S2: Organic acids levels in cecal content after the treatment for 12 weeks among the control (CON), conventional quercetin (CQN), and EubioQuercetin (EQN) groups; Table S3: Intestinal gene expression associated with aging score after the treatment for 12 weeks.
